# Is clinical, musculoskeletal pain associated with poorer logical reasoning?

**DOI:** 10.1097/PR9.0000000000000929

**Published:** 2021-05-07

**Authors:** Helena Gunnarsson, Jens Agerström

**Affiliations:** Department of Psychology, Faculty of Health and Life Sciences, Linnaeus University, Växjö, Sweden

**Keywords:** Clinical pain, Experimental pain, Logical reasoning, Cognitive functions

## Abstract

**Introduction:**

It has been hypothesized that pain disrupts system 2 processes (eg, working memory) presumed to underlie logical reasoning. A recent study examining the impact of experimentally induced pain on logical reasoning found no evidence of an effect.

**Objectives:**

The aim of this study was to examine whether clinical pain, which is qualitatively different from experimental pain, would lower the ability to reason logically.

**Methods:**

Ninety-six participants completed a questionnaire containing 3 different logical reasoning tasks (the cognitive reflection test, the belief bias syllogisms task, and the conditional inference task), questions about pain variables (present pain intensity, pain intensity during the last 24 hours, the influence of pain on daily activities, pain duration, and pain persistence), questions about other pain-related states (anxiety, depression, and fatigue), and pain-relieving medication. Correlations between the logical reasoning tasks and the pain variables were calculated.

**Results:**

For 2 of the 3 logical reasoning tasks (the cognitive reflection test and the belief bias syllogisms task), clinical pain was unrelated to logical reasoning. Performance on context-free logical reasoning showed a significant negative correlation with present pain intensity, but not with the other pain variables.

**Conclusion:**

This finding that logical reasoning ability is largely unrelated to clinical pain is highly consistent with previous research on experimentally induced pain. Pain should probably not constitute a significant barrier to logical reasoning in everyday life.

## Abstract 2

Clinical pain does not seem to be considerably related to logical reasoning ability, which seems consistent with the effect of experimental pain on logical reasoning.

## 1. Introduction

Clinical pain has been found to have a debilitative effect on numerous cognitive functions.^5,6,24,31^ A recent study examined the impact of experimentally induced pain on logical reasoning.^3^ It was hypothesized that pain would impair logical reasoning through a disruption of type 2 processing, which is characterized by effortful, slow, structured, analytical, and flexible cognitive processing, as opposed to type 1 processing, which involves automatic, nonconscious processing.^14,24,25^ Specifically, the authors theorized that to prioritize avoidance of harm and facilitate pain reduction, pain would disrupt attention and, more importantly, working memory processes,^6,8^ which are considered to play a major role in type 2 processing.^19^ This would also be consistent with limited capacity models where pain and demanding cognitive tasks are assumed to compete for limited cognitive resources.^15^

The reflective type 2 system has been theorized to play a major role in logical reasoning by checking and possibly revising intuitive answers.^19,26^ Indeed, activation (deactivation) of type 2 processing has been empirically shown to improve (impair) logical reasoning.^14^

The study by Attridge et al.^3^ examined whether pain would impair performance on 3 different logical reasoning tasks presumed to draw substantially on type 2 processing: the cognitive reflection test (CRT), the belief bias syllogisms task, and the conditional inferences task. For example, the CRT contained problems designed to elicit an intuitive answer (type 1) that is incorrect. For the answer to be correct, the immediate answer had to be cognitively overridden by further reflection and deliberation to find the correct answer (type 2). Contrary to their hypothesis, Attridge et al. found no significant effect of experimental pain on logical reasoning.^3^ Although an experimental method achieves a high level of control of extraneous variables, the short duration and the unthreatening nature of experimentally induced pain makes it rather different from real-world pain, where the duration, the representation in the brain,^2,11^ and the negative emotions coexisting with clinical pain constitute important differences.^1^ For example, clinical pain coexists with negative feelings, such as anxiety and depression.^10,27,34,38,42^ Clinical pain could be categorized into subgroups with acute pain (pain <3 months) and chronic pain (pain >3 months) being the 2 subgroups that are most frequently used in conjunction with benign pain.^41^ Although the most prevalent distinction used in the pain literature concerns chronic vs acute pain, another way to explore different pain states could be through a continuous scale when considering different pain variables. The reason for this is that differences in the duration and persistence of pain could affect the brain differently, even in patients within the chronic pain group.^4,22,24^

Impaired logical reasoning could be detrimental to both the society and the individual with pain. If it is found that clinical, musculoskeletal pain impairs logical reasoning, the effects could have nontrivial consequences for life domains, such as education, problem solving, and risk assessment. In this study we wanted to conceptually replicate the research by Attridge et al.^3^ Instead of examining the link between experimentally induced pain and logical reasoning, we examined whether logical reasoning would be associated with clinical pain (defined as a naturally occurring pain without experimental provocation).^16^ The focus on clinical pain makes a novel contribution to the largely unexplored link between pain and logical reasoning.

## 2. Materials and methods

### 2.1. Study population

Participants with fibromyalgia syndrome were recruited from the patient union “Fibromyalgiförbundet” through information about the study presented on the union Web site. From the patient union we received 109 answers, and questionnaires were delivered to these participants. Of these questionnaires, 78 were returned. Eighteen participants were also recruited through information pamphlets about the study in waiting rooms at primary care centers in the southeast of Sweden. These 18 participants received and returned their questionnaires. No participants were excluded because of lack of understanding of the task. In total, 96 individuals participated. Participants with clinical musculoskeletal pain were chosen because these diagnoses are common in society and are responsible for high societal costs.^29,36^ Inclusion criteria were perceived pain from muscles or joints. Exclusion criteria were younger than 18 years and not being fluent in Swedish. Pain-relieving medications were used daily in 66 participants. Medications consisted of analgesic drugs, such as paracetamol, tramadol, and altermol. Anti-inflammatory drugs, opioids, muscular relaxants, antiepileptics, and antidepressant drugs were also used. A daily combination of 2 or 3 pain-relieving drugs was used by 35 participants, and the rest of the participants used only one pain-relieving drug daily. The patients recruited from the patient union had received their fibromyalgia diagnosis from a physician, and patients recruited from waiting rooms at primary care centers had received their diagnosis from either a physician or a physiotherapist. All participants reported their diagnosis in the questionnaire. In the Brief Pain Inventory-Short Form (BPI-SF), they were also asked to shade the areas where they felt pain on a human figure. The shaded areas matched the diagnosis in all participating individuals (Table 1). All participants signed an informed consent form, and the regional ethics review board in Linköping approved the study (code: 2019-02071).

**Table 1:** *International Classifications of Disease* (ICD-10) codes for each pain diagnosis included and the number of participants.

	Fibromyalgia syndrome (M797)	Other (M19; primary arthrosis), (R52; pain), (M48; spinal stenosis), (M353; polymyalgia rheumatica), (S801; contusion), (M10; gout), (M543; sciatica) (M542; cervical pain), (M17; knee arthrosis), and (S46; tendon injury in the shoulder).
N	78	18

### 2.2. Procedure

The individuals wanting to participate in the study, emailed the study director for further information, and following that information, all of them provided an address to send the questionnaire. A questionnaire was sent home to the participant with instructions to complete the questionnaire individually, during a single test session, and in a quiet location. It was further stressed that they were not allowed to seek help from another person or the Internet. After having answered the questionnaire, they sent it back to the study director in an envelope, free of postage.

### 2.3. Measures: independent variables

#### 2.3.1. Reported pain intensity and influence on daily activities

In the questionnaire, the participants were asked to estimate their present pain on a visual analogue scale (VAS). On a 10-cm perpendicular line, each participant placed a mark between the 2 endpoints: no pain and worst imaginable pain. Based on the distance on the 10-cm line, the participants received a score from 0 to 10. The VAS is a subjective scale, which means that other factors external to the immediate pain sensation could influence every pain rating.^30^ Nevertheless, the VAS is a traditional method of pain measurement, and it has several benefits; it is simple, effective, and widely used in both research and in clinical practice.^28^ It has been regarded to be a valid scale to measure both experimental and chronic pain.^32^ Using the BPI-SF, the participants estimated their clinical pain intensity during the last 24 hours and the influence of pain on daily activities during the last 24 hours. The BPI-SF has been widely used and good reliability and validity has been reported.^12^ The ratings were made on a scale ranging from 0 (no pain or interference) to 10 (worst pain or interference) for BPI-SF intensity and BPI-SF interference, respectively.

#### 2.3.2. Pain duration

In the questionnaire, participants reported when they first experienced the pain they were currently suffering from. They answered how often the pain was recurring if it was not persistent. The number of days since the first pain episode was used as the measure of pain duration if the pain was frequently recurring, at least several times a week. If the pain was not frequently recurring several times a week, the number of days of the last pain episode was used as the duration measure.

#### 2.3.3. Pain persistence

To assess pain persistence, the participants reported how much time they were in pain during the day as a percent.

#### 2.3.4. Fatigue

How much of the time the participants felt tired during the day as a percent.

#### 2.3.5. Depression and anxiety

The Hospital Anxiety and Depression Scale (HADS) was used to measure any presence of anxiety or depression.^43^ In HADS, 7 items measured anxiety and 7 items measured depression. The highest possible score was 21 for each subscale and the lowest score was zero. The validity and the internal consistency of the HADS scale have been reported to be good (α = 0.6).^9^

### 2.4. Outcome measures: logical reasoning

We used the same measures as in the earlier experimental study,^3^ but we used fewer items because our experience is that when measuring cognitive abilities in clinical pain samples, participants often terminate their participation or decline to participate if they are presented with longer questionnaires.^22,23^

The logical reasoning measures were translated to Swedish by a translator with a master's degree in English (linguistics) and substantial experience in translating similar texts. The Swedish versions were pretested on a handful of participants to ensure that the meaning of the items was clear and concise. The translated versions are available in Figshare: https://figshare.com/articles/journal_contribution/Untitled_Item/14237015.

The order in which the dependent measures (the CRT, the belief bias syllogisms task, and the conditional inference task) appeared in the questionnaire was counterbalanced.

#### 2.4.1. The cognitive reflection test

The CRT^21^ contains problems designed to elicit an immediate, intuitive answer (type 1) that is incorrect, but this faulty answer could be overridden by slower type 2 processing that involves further reflection and deliberation to find the correct answer. We used the original version of the CRT with 3 items.^40^ The CRT has shown moderate correlations with rational thinking ability and cognitive ability. Furthermore, the 3-item CRT was able to predict substantial unique variance, when compared with intelligence tests, cognitive ability, and executive functioning tests.^40^ Comparing our results with normative data, 45% participants in our sample solved none of the questions correctly and 7.4% solved all the questions correctly, whereas 42.2% solved none of the questions correctly and 12.9% solved all items correctly in a reliability study.^7^

#### 2.4.2. The belief bias syllogisms task

As a measure of the ability to reason independently of previous beliefs, the belief bias syllogisms task was used.^35^ It requires participants to disregard their spontaneously activated default beliefs (type 1) and instead use analytical thinking to determine whether conclusions would follow logically (type 2) from various syllogisms. Because we wanted a short version, 6 items from the original scale were chosen. The belief bias syllogisms task consisted of 24 thematic syllogisms of different congruency and validity (4 incongruent, believable-valid; 4 neutral, neutral-invalid; 4 congruent, unbelievable-invalid; 4 congruent, believable-valid; 4 neutral, neutral-valid; and 4 incongruent, unbelievable-valid). To be able to incorporate these different components, one item from each group was used. For each one, the participant had to decide whether the syllogism was logically valid by circling “yes” or “no.” The instructions informed the participants to consider only the information presented in each item and to limit themselves to the information presented in the item only. The subjects were correct in 99% of the cases compared with earlier performances where subjects were correct in 97% of the cases when belief agreed with logic in an earlier study.^17^ Moreover, in our study 55% were correct compared with 43%^17^ when belief conflicted with logic. The percentage of subjects accepting conclusions in the invalid items was 68% in our study compared with 69% in the earlier study.^17^ The percentage of subjects accepting conclusions in the valid items was 87% in our study compared with 91% in the earlier study.^17^

#### 2.4.3. The conditional inference task

In the conditional inference task, the participants determined whether conclusions drawn from conditional rules or premises are logically valid or invalid. It consists of 4 forms of context-free abstract inferences, the modus ponens (true antecedent implies true consequent), the denial of the antecedent (DA; false antecedent implies false consequent), the affirmation of the consequent (AC; true consequent implies true antecedent), and the modus tollens (false consequent implies false antecedent).^18^ Because we needed a short version, 8 items in total were selected with 2 items from each group (2 modus ponens, 2 DA, 2 AC, and 2 modus tollens). Our test with 2 items from each group showed the same performance pattern as in another study^37^ where most people performed well on modus ponens (97% compared with 94% in this study) and modus tollens (72% compared with 59% in this study). Regarding the DA (45% compared with 56% in this study) and AC (37% compared with 18% in this study) inferences, the performance was substantially lower. For each item, the participants had to decide whether the presented item was true or false by circling “yes” or “no.”

#### 2.4.4. Power analysis

An a priori power analysis using G* Power was performed. We wanted a power level of 85% to be able to detect a moderate effect size (r = 0.30), with an alpha level of 0.05 (2-tailed test). A minimum sample of 93 participants would be required, following these criteria in the context of a point-biserial correlational model.

#### 2.4.5. Data analysis

Correlation analyses were performed using jamovi version 1.0.7 solid.^33,39^ When inspecting the data, most variables did not approximate a normal distribution, and therefore nonparametric Spearman correlational analyses were performed.

## 3. Results

Descriptive statistics are presented in Tables 2 and 3. In Table 2, the sociodemographic characteristics reveal a mean sample age of 49 years, 90% of the participants in the study were women, 91.7% had a higher education than elementary school, and 68.7% used daily pain-relieving medication. Table 3 shows mean values including pain intensity on the VAS and the BPI-SF (6.15 and 6.09), pain duration (6206 days), pain persistence (86.4%), and pain influence on daily activities (6.44).

**Table 2:** Sociodemographic characteristic of the study participants.

Variable	N = 96
Age in year, mean (SD)	49 (12)
Sex: female n (%)	90 (94)
Education level n (%)	
Elementary	8 (8.3)
High school	48 (50.0)
University	40 (41.7)
Daily intake of pain-relieving medication, n (%)	66 (68.7)

**Table 3:** Descriptive statistics with means, SDs, and minimum and maximum values.

	Mean (SD)	Minimum	Maximum
Pain intensity VAS	6.15 (2.28)	0	9.8
Pain duration (days)	6206 (5029)	1	22630
Pain persistence (%)	86.4 (22.6)	0	100
Pain intensity BPI-SF	6.09 (1.76)	0	10
Pain influence on daily activities BPI-SF	6.44 (2.38)	0	10
Fatigue (%)	74.01 (27.34)	0	100
Anxiety	9.96 (5.48)	0	21
Depression	7.79 (4.98)	0	21
The CRT	0.78 (0.9)	0	3
The belief bias syllogisms task	3.97 (1.18)	0	6
The conditional inference task	4.53 (1.02)	0	8

BPI, brief pain inventory; CRT, cognitive reflection test; VAS, visual analogue scale.

Table 4 reports Spearman correlations with the Bonferroni adjusted alpha level (*P* < 0.006). As shown, we find a negative correlation between present pain intensity on the VAS and the ability to perform context-free logical reasoning tasks (the conditional inference task), with a small to moderate effect size (rho = −0.288, r^2^ = −0.083, *P* < 0.006). Thus, higher present pain intensity is associated with worse context-free logical reasoning performance. However, no other pain variables (pain intensity during the last 24 hours, the influence of pain on daily activities during the last 24 hours, pain duration, and pain persistence) or pain-related variables (pain-relieving medication, HADS depression, HADS anxiety, and fatigue) showed any significant correlation with the logical reasoning measures (the CRT, the belief bias syllogisms task, and the conditional inference task). To assure that the association between pain and logical reasoning did not differ for the different patient groups, we also performed additional analyses that only included the fibromyalgia patient group. Because these results were virtually identical, we decided not to report them.

**Table 4:** Correlation matrix.

		The visual analogue scale	BPI-SF intensity	BPI-SF influence of daily activities	Pain duration	Pain persistence	HADS anxiety	HADS depression	Fatigue	Age	Gender
The cognitive reflection test	Spearman rho	−0.048	−0.086	−0.168	−0.151	0.056	−0.168	−0.142	−0.062	−0.058	−0.010
	*P*	0.644	0.413	0.110	0.154	0.595	0.108	0.175	0.559	0.577	0.926
The belief bias syllogisms task	Spearman rho	−0.060	−0.084	0.023	−0.082	−0.026	0.050	−0.077	−0.069	−0.052	−0.035
	*P*	0.562	0.423	0.828	0.438	0.809	0.630	0.458	0.509	0.614	0.074
The conditional inference task	Spearman rho	−0.288*	−0.168	−0.208	0.134	0.022	−0.112	−0.184	−0.104	0.149	−0.038
	*P*	0.006	0.115	0.052	0.213	0.842	0.297	0.084	0.335	0.162	0.725
The visual analogue scale	Spearman rho		0.669*	0.477*	0.162	0.307*	0.316*	0.436*	0.441*	−0.240*	0.259
	*P*		<0.001	<0.001	0.120	0.003	0.002	<0.001	<.001	0.018	0.012
BPI-SF intensity	Spearman rho			0.530*	0.194	0.414*	0.394*	0.437*	0.428*	−0.123	0.268
	*P*			<0.001	0.064	<0.001	<0.001	<0.001	<0.001	0.236	0.009
BPI-SF influence of daily activities	Spearman rho				0.128	0.345*	0.717*	0.734*	0.456*	−0.232*	0.251
	*P*				0.226	<0.001	<0.001	<0.001	<0.001	0.025	0.016
Pain duration	Spearman rho					0.291*	0.004	−0.004	0.169	0.285*	0.279
	*P*					0.005	0.970	0.973	0.110	0.006	0.007
Pain persistence	Spearman rho						0.127	0.205	0.311*	0.093	0.262
	*P*						0.227	0.050	0.003	0.375	0.012
HADS anxiety	Spearman rho							0.773*	0.409*	−0.415*	0.225
	*P*							<0.001	<0.001	<0.001	0.030
HADS depression	Spearman rho								0.486*	−0.293*	0.207
	*P*								<0.001	0.004	0.046
Fatigue	Spearman rho									−0.295*	0.328*
	*P*									0.004	0.001

Correlation matrix showing Spearman correlations between the outcome variables (the cognitive reflection test, the belief bias syllogisms task, and the conditional inference task) and the independent variables (visual analogue scale, BPI-SF pain intensity, BPI-SF pain influence of daily activities, HADS anxiety, HADS depression, pain duration, pain persistence, fatigue, and age).

* Denotes significant correlations at the *P* < 0.006 level (Bonferroni adjusted).

## 4. Discussion

This study found weak support for the hypothesis that clinical pain is associated with poorer logical reasoning. Only one significant correlation was found. Higher pain intensity on the VAS was associated with a decreased ability in context-free, logical reasoning performance. The effect size was in the small to moderate range. We found no correlation between any of the other clinical pain variables and the CRT, the belief bias syllogisms task, or the conditional inference task.

Only one previous study has examined the effect of short-lasting, experimental pain on logical reasoning but found no support for the hypothesis that pain would affect logical reasoning.^3^ Overall, our study showed highly similar results. For 2 of the 3 logical reasoning measures (the CRT and the belief bias syllogisms task) there was no relationship between pain and logical reasoning. In the third, we found a relationship, where higher present pain intensity was associated with decreased context-free logical reasoning performance with a small to moderate effect size.

Pain intensity has been linked to worse executive functioning.^8^ Our finding that higher pain intensity is associated with worse performance on context-free logical reasoning is consistent with this. Clinical pain of different durations has also been associated with more errors in higher-level cognitive numerical and everyday decision-making tasks, although with a small effect size.^4^ Context-free logical reasoning ability involves higher cognitive functions for controlled and energy-consuming type 2 processing, and maybe this is the reason why a significant correlation was found between these variables. The CRT and the belief bias syllogisms task did not correlate significantly with the pain variables. Compared with performance on the other tasks, the CRT scores seem to have been more restricted in range (mean = 0.78 of a maximum score of 3), possibly making it more difficult to detect a correlation between performance on this task and pain. Nevertheless, the CRT tasks are designed to elicit an automatic and incorrect response resulting from type 1 processing when reading the problem, and it is the role of reflective type 2 processing to detect the error of that fast response, facilitating a correct answer.^40^ Possibly, few participants in the sample were able to override the fast and incorrect response when attempting to solve this task because of a lack of type 2 processing. This would be consistent with previous research showing that chronic pain is associated with a decreased ability to inhibit automatic responses.^8^

Regarding the belief bias syllogisms task, the mean value for the sample (3.97 of a maximum score of 6) showed that there was more variance in the sample, and therefore it seems that participants were able to engage in deductive reasoning to varying extents. The lack of significant correlations between performance on this task and the pain variables is consistent with the results of the study by Attridge et al. examining the effect of experimental pain on logical reasoning ability.^3^

In relation to normative data for performance on the logical reasoning tests, it seems as if our sample performed at fairly comparable levels. This suggests that there was nothing unusual about our sample regarding logical reasoning ability.

The observed relationship between present pain intensity and context-free logical reasoning could have some limited effects on specific parts of life, eg, examination results relying on context-free logical reasoning. Because the ability to engage in context-free logical reasoning seems to be more affected at higher pain intensities and no association between pain-relieving medication and any decreased ability in logical reasoning was found, it might be a wise decision to use pain-relieving medication in situations where context-free logical reasoning could be required.

In our pain sample, most patients (81%) suffered from fibromyalgia syndrome, although other musculoskeletal pain states, such as cervical pain and arthrosis, were also present (19%). Thus, the pain sample was heterogenous. However, this is the reality of clinical, musculoskeletal pain states. This could be seen both as a strength and limitation of this study. The strength is that the results could be more easily generalized to the real pain population with varying pain durations and diagnoses. The limitation consists of potential confounding variables inherent in heterogenous pain samples. For confounding variables, it would have been desirable to have more homogenous pain diagnoses in the sample. However, the complex and dynamic nature of pain perception means that all pain samples will be heterogenous because every pain experience is unique between individuals and even in a duration-dependent manner within the same individual.^20^

A related limitation of this study could be that we used mixed cohorts (acute and chronic pain). However, we believe that one viable approach to the study of clinical pain is to conceptualize its components (eg, duration and persistence or recurrence) as falling on a continuum and examine how each of them relate to cognitive outcomes.^22,24^ This approach should capture differences that exist between different pain categories (acute vs chronic) and within the same pain category (eg, chronic) regarding fundamental pain components, such as its duration (eg, 1 week vs 5 months vs 10 years). Arguably, this noncategorical approach should entail a more sensitive measurement of the pain component in question, yet with the drawback of increased sample heterogeneity and associated error variance.

Ideally, any study wishing to examine how clinical pain influences cognition should include a pain-free, otherwise identical, control group. However, this would be difficult to achieve in any study on clinical pain as experimental pain induction is not possible. Even if meticulous matching is used to control for all possible group differences that could confound an association between clinical pain and cognition, there are always unobserved factors that could act as confounders. Although there are certainly potential confounding variables in a study examining correlations between naturally occurring pain variables and logical reasoning among pain patients only, it avoids unobserved between-group differences that are typically introduced by a nonequivalent control group in a nonrandomized study.

In this study, a shortened version of the 3 different tests (the CRT, the belief bias syllogisms task, and the conditional inference task) were used because our earlier experiences in conducting questionnaire testing in clinical pain samples have shown that patients faced with long questionnaires tend to terminate the questionnaire in advance or decline to participate in the study. We did not want to expose the patients to a situation where cognitive overload and fatigue would influence the logical reasoning results. Of course, this could be a potential limitation to the study because the shortened tests have not been validated in a previous study. It is possible that they provided a less reliable measurement of logical reasoning.

Although logical reasoning is traditionally considered as a prototypical example of a task that requires effortful type 2 processing, this theoretical claim has not gone uncontested. In fact, there is some research suggesting that people can process logical principles in classic reasoning tasks intuitively and without much deliberation.^13^ One possible reason why we do not find a reliable, overall relationship between clinical pain and logical reasoning is that it involves both system 1 and system 2 processing.

In conclusion, the current research contributes to the research field on the relationship between clinical musculoskeletal pain and logical reasoning abilities. To the best of our knowledge, this is the first study to explore the link between clinical musculoskeletal pain and logical reasoning. Overall, our results were consistent with previous research on the link between experimental pain and logical reasoning,^3^ suggesting that as with experimentally induced pain, logical reasoning is largely unaffected by clinical pain.

## Disclosures

The authors have no conflicts of interest to declare.
